# “Better start”: promoting breastfeeding through demarketing

**DOI:** 10.1186/s12889-023-16561-3

**Published:** 2023-08-31

**Authors:** Mohammed Salem, Myriam Ertz

**Affiliations:** 1 Business Department, University College of Applied Sciences, Remal, P.O. Box 1415, Gaza, Gaza Strip Palestine; 2 Department of Economics and Administrative Sciences, Université du Québec à Chicoutimi, Saguenay, Canada

**Keywords:** Demarketing strategies, Breastfeeding, Attitude, Deconsumption, Palestine

## Abstract

**Background:**

This paper explores how demarketing strategies impact women’s breastfeeding attitudes, intentions, and behaviors under the moderation of time pressure and breastfeeding knowledge.

**Methods:**

A cross-sectional questionnaire-based survey among 369 respondents is used to test the proposed hypotheses. The study's population includes all breastfeeding women in Palestine. Snowball and convenience sampling were used to choose study participants through personal connections and social media. Every respondent was encouraged to share the survey with their social media contacts.

**Results:**

The data results confirm the positive effects of promotion, place, price, and product demarketing, respectively, on women’s attitudes, intentions, and behavior toward breastfeeding. These effects were reinforced by reduction in time pressure and breastfeeding knowledge. Furthermore, demarketing effects are stronger for younger, more educated, unemployed, and lower-income women.

**Conclusion:**

The study is a primer on promoting breastfeeding instead of formula by means of demarketing strategies.

## Introduction

Although marketing is a critical component of organization management, “demarketing” is less known. It aims to dissuade customers from consuming or buying specific products, either because they are harmful or simply because consumer demand exceeds supply [1]. As such, it has often been used from a social marketing perspective. Yet, demarketing is being rediscovered with the sustainability and ethical tide in marketing (e.g., [2, 3]). It appears essential in a constantly changing environment where socioecological concerns are increasingly important [4]. The core of demarketing is to improve society's well-being and safety by lowering demand for a particular product due to its harmful features. Over the years, mounting evidence suggested that breastmilk substitutes might fall into the category of relatively harmful products.

Breastfeeding poses challenges for mothers, including delayed milk supply, physical discomfort, and inadequate milk production, which can leave them feeling overwhelmed and worried about their baby's nutrition [5]. Throughout history, women have used various methods to address breastfeeding difficulties, such as wet nursing, cross-nursing, and milk donations. However, these practices have faced societal unease, infection concerns, and religious condemnations, as they were seen as intrusive to the mother-infant relationship [6]. Bottles and formulas quickly replaced these historical practices due to their convenience, affordability, and accessibility [7]. Despite the ease of formula feeding, the benefits of breastfeeding tend to outweigh the challenges.

Extensive research [8, 9] has emphasized the advantages of breastfeeding for both mothers and infants. Breastfeeding provides ideal nutrition, immunization, and safe nourishment for babies. It reduces the risk of infections and diseases, promotes mother-infant interaction, and contributes to psychological and intellectual development in infants.

In contrast, breastmilk substitutes have sometimes been associated with worldwide health risks. Formula feeding has been linked to the growth of obesity, metabolic diseases, and infections in children [8]. Moreover, formula feeding is significantly more expensive and poses additional risks in developing countries, where clean water and proper hygiene are often lacking. Contaminations in powdered infant formula have resulted in severe illnesses and infant mortality, making it a significant global health risk. Formula feeding and suboptimal breastfeeding contribute to a substantial number of child deaths worldwide [10, 11].

Yet despite this increasingly common knowledge on the benefits of breastfeeding and the risks associated with bottle-feeding, especially in developing countries, breastfeeding remains stagnating. According to earlier research [12], only 39% of children globally are breastfed exclusively for four months, with a much lower proportion being breastfed for the entire six months suggested.

The World Health Assembly adopted the International Code of Marketing of Breastmilk Substitutes to promote breastfeeding and discourage breastmilk substitutes. This worldwide public health policy aims to ensure that women are not prevented from breastfeeding and that breastmilk substitutes are used safely when necessary. The Code emphasizes marketing limitations on breastmilk substitutes and ethical guidelines for feeding bottles and teats. These initiatives demonstrate a growing effort to restrict or demarket breastmilk substitutes in favor of breastfeeding promotion [13].

Promoting breastfeeding through demarketing in Palestine is crucial due to concerns regarding the unethical marketing practices surrounding formula milk. While specific data on the situation in Palestine is limited, common challenges worldwide include aggressive marketing tactics directly targeting mothers and healthcare professionals, inadequate breastfeeding support, misleading health claims by formula milk companies, lax regulation and oversight, and potential influence on healthcare professionals. Conducting research specific to Palestine, including literature review and primary data collection, would provide a more accurate understanding of the issue and aid in developing targeted demarketing strategies to promote breastfeeding and counter the unethical marketing of formula milk.

This study explores how marketing strategies can encourage the deconsumption of breastmilk substitutes and promote breastfeeding. Drawing on the demarketing framework [14], the study investigates the impact of demarketing strategies on the four Ps of marketing (product, price, place, promotion) on consumer attitudes, intentions, and behavior towards breastfeeding. Additionally, the study explores the moderating effects of time pressure and breastfeeding knowledge on the influence of demarketing strategies. Time pressure refers to the perception of a time restriction to digest information to make decisions. This paper contributes to the field by examining how demarketing strategies can shape consumer attitudes and behavior toward breastfeeding in Palestine. It also investigates the moderating role of time pressure and breastfeeding knowledge.

The remainder of this paper is structured as follows: the relevant literature is studied, the methodology used is explained, the findings are evaluated and debated, and finally, conclusions and recommendations are offered.

## Conceptual framework and hypotheses development

The theoretical foundations of the demarketing approach are provided in this section. Each component of demarketing is explored, and the hypotheses are put out in light of current theoretical and empirical studies.

### Attitude toward breastfeeding

An attitude is a long-lasting positive or negative sentiment (or taught dispositions) towards a person, object, or issue [15]. In this study, a consumer's attitude toward breastfeeding is described as the level of their acquired predisposition toward breastfeeding, whether favorable, negative, or neutral. Both theoretical and empirical data from earlier studies validated the hypothesized model's prediction that consumers' attitudes toward breastfeeding are influenced by demarketing strategies (i. e., product, price, place, and promotion).

The theory of planned behavior (TPB) [16] and the theory of reasoned action (TRA) [17] were founded on the notion that the most significant predictor of one's intention to carry out a behavior is one's attitude toward it. In addition, numerous empirical studies in the fields of psychology, consumer behavior, and marketing have shown the significance of attitude in comprehending behavioral intention [18].

### Demarketing theory

Kotler [14] asserts that the terms "demarketing" and "demand reduction" are interchangeable. According to Kotler and Levy ([19], P. 75), demarketing is "that aspect of marketing that deals with discouraging customers in general or a particular class of customers, on either a temporary or permanent basis." This concept is used in the current study and is broadly recognized by marketing academics and practitioners. Organizations and/or governments adopt demarketing by changing the components of the marketing mix (i.e., product, price, place, and promotion). Demarketing is necessary for four circumstances [14]: 1) Managing an already existing scarcity; 2) Preventing possible shortages; 3) Reducing harm to people; and 4) Reducing harm to the environment or special resources. With rising socioenvironmental concerns, demarketing is gaining traction again in order to curb the consumption of finite resources and reduce harm to human well-being.

Demarketing does come with a few warnings, though [14]: First, demarketing efforts may increase consumer demand for the targeted goods (e.g., banning a book or movie). Second, demarketing initiatives might lead to the formation of a criminal elite that would profit from the artificial scarcity (e.g., the United States’ “prohibition era” when alcohol was outlawed). Third, liberals and libertarians may view the government's or even corporate interferences with certain fundamental human rights (e.g., the rights to purchase and consume) as a major point of contention.

However, it might be argued that in some specific contexts, such as Palestine with its unique social (high density and spread of diseases), economic (economic blockade, limited income sources, and need to curb costly imports to retain a balanced budget), and political situation (conflicts and wars), demarketing techniques might appear as a least-worse solution to promote favorable attitudes, intentions, and behavior around breastfeeding. According to Kotler [14], demarketing is most effective when there is a strong public agreement that the consumption of a certain commodity or service should be reduced. This study aims to do just that by soliciting customer feedback on various demarketing actions.

Since the advent of social marketing, ethical marketing, and sustainability in marketing, empirical research on the demarketing of breast milk substitutes has increased (e.g. [1, 20],). However, neither of these earlier studies considered time pressure, breastfeeding knowledge, or demarketing tactics. Additionally, we looked at the attitudes, intentions, and behaviors associated with breastfeeding connected to demarketing strategies. This study suggests that time pressure reduction and breastfeeding knowledge are crucial factors with the potential to boost demarketing managerial efforts to promote breastfeeding, in contrast to prior research and to advance the state-of-the-art. Last but not least, no study examined the demarketing of breastmilk substitutes in developing economies, particularly in the unique setting of Palestine, except for a few studies (e.g. [1],).

Based on the above, demarketing is a marketing approach that discourages the demand for a specific product or service. In this paper, demarketing involves strategies to reduce the consumption of formula milk and encourage breastfeeding instead. This would include awareness campaigns, educational materials, and policy advocacy to create a supportive environment for breastfeeding. The objective is to shift societal norms and behaviors towards breastfeeding for improved infant health and well-being.

### Relation between the demarketing mix and consumer response

This study focuses on corporate demarketing approaches, not government demarketing initiatives. We assume that the demarketing aspects of the product (H1), pricing (H2), place (H3), and promotion (H4), respectively, have a direct impact on consumers' attitudes toward breastfeeding.

The product relates to marketing activities to lower product sales volume. Salem et al. [21] assert that firms attempt to restrict supply lines, reduce service, and eliminate productive actions that attract customers to buy the product as part of the demarketing notion. Additionally, it is generally known that happy consumers increase profitability since they tend to acquire more product units and make frequent long-term repurchases [22]. Contrarily, unhappy clients bring in less money since they make fewer purchases and consume less of the company's products. Additionally, complaints and negative word of mouth are frequently a result of their dissatisfaction [23]. A dissatisfied client would therefore be more prone to develop deconsumption attitudes, intentions, and behaviors if contentment boosts consumption and dissatisfaction decreases it. Additionally, promoting and encouraging the adoption of substitute products is an essential demarketing tactic [21]. Empirically, it has been demonstrated that product modification favors the demarketing of breast milk substitutes [1]. In light of the preceding, we postulate:

- ***H1:*** Product modulation positively affects consumers’ attitudes toward breastfeeding.

The demarketing of breast milk substitutes may be easily solved through price setting. The demarketing pricing approach aims to reduce present demand by raising prices. However, pricing affects the attitudes and actions of customers. Customers who believe prices are too expensive are likely to have a bad opinion of the product and have little interest in purchasing it [24]. In light of this, demand will decline as prices rise. However, determining pricing is one of the trickiest decision-making processes in demarketing [25]. Therefore, the demarketing pricing strategy seeks to reduce but not eliminate product demand by raising prices. For instance, it has been demonstrated in Palestine that price modulation favors the demarketing of breast milk substitutes [1]. Therefore, we propose the following hypothesis:

- ***H2:*** Price modulation positively affects consumers’ attitudes toward breastfeeding.

According to previous studies, place/location is one of the crucial elements in demarketing. Both the place of consumption and the place of purchase may be modified, and both factors may have a demarketing impact [21]. Ajzen [26] asserts that restricting the areas where a behavior can be practiced might hinder its promotion and negatively affect intentions. Restrictions on consumption are a crucial barrier to consumption, hence they should have a direct effect on intentions to consume. Limitations on purchasing would also produce a similar outcome, reducing the impact of opportunity cost (e.g. [27],). When used in a demarketing context, this suggests that investing more time and effort in product research would reduce the time available for other activities like leisure, household chores, or self-maintenance. In addition, it slows down the demarketing of breast milk substitutes when place modification is used [1]. Therefore, we suggest the following:

- ***H3:*** Place modulation positively affects consumers’ attitudes toward breastfeeding.

The demarketing approach uses promotions to persuade consumers to buy products they won't use and anti-promotional tactics to justify usage. Nguyen et al. [28] point out that social marketing campaigns focus on educating the public about the benefits of breastfeeding behavior and the necessity of protecting both mothers and infants as one of its primary objectives. Because consumer promotion has a direct negative influence on intentions, research supports its relevance in demarketing [29]. Consumers will consider demarketing breast milk substitutes since they have a greater view of promotion and communication attempts to deconsume [1]. Consequently, we expect the following:

- ***H4:*** Promotion modulation positively affects consumers’ attitudes toward breastfeeding.

### The moderating effect of reduction of time pressure

A temporary aspect of a situation is referred to as time. Behavioral changes throughout a person's life limit the amount of time available to them. As a result, time pressure refers to the perception of a time restriction on which to digest information in order to make decisions [30]. Consumers in a hurry may only think about what products to buy and where to acquire them. Under time pressure, customers may become more stressed and face purchasing risk [31]. Furthermore, time pressure negatively influences impulsive buying since customers are frustrated when they don't have enough time to shop or browse [32]. In other words, time pressures limit consumer search activities since they make it harder for people to digest information. This may cause some customers to act quickly, while others may postpone their decisions. Consequently, those strategies will be less affected by individuals perceiving modulations on breastmilk substitutes (i.e., demarketing strategies on the 4 Ps). This is because they will be less likely to invest mental and psychological efforts to process the information since this would require too much time and effort. Based on the above, we propose the following hypothesis:

- ***H5:*** Reduction of time pressure amplifies the effect of (a) product, (b) price, (c) place, and (d) promotion on consumers’ attitudes toward breastfeeding.

### The moderating effect of breastfeeding knowledge

Individuals with appropriate contextual knowledge of exclusive breastfeeding do better in practice, but those with little knowledge perform worse. Changes in attitudes toward breastfeeding are frequently considered to mainly depend on knowledge and belief of the phenomena in question [33]. Mothers' attitudes are essential in the exclusive breastfeeding practice, which is primarily explained by maternal knowledge. A lack of good attitudes toward exclusive breastfeeding and a lack of knowledge may result in a low likelihood of timely exclusive breastfeeding beginning and continuation [34]. Furthermore, education and prior experience may serve as valuable knowledge sources and help shape one's attitude toward performance. A multiparous mother's previous breastfeeding experience might substantially impact her attitude toward exclusive breastfeeding [35]. As a result, it was essential to look into the effect of exclusive breastfeeding knowledge and past experience on mothers' attitudes on breastfeeding. Therefore, we propose the following hypothesis:

- ***H6:*** Breastfeeding knowledge amplifies the effect of (a) product, (b) price, (c) place, and (d) promotion on consumers’ attitudes toward breastfeeding.

### Behavioral intention

The cognitive activity, known as behavioral intention, describes how a buyer intends to purchase a certain brand. In the theories of reasoned action and planned behavior [16], intention significantly influences the choice to purchase. Intentional measures, instead of behavioral ones, may be more effective in eliciting customers' ideas because they may have purchased due to restrictions rather than actual preferences [36]. As a result, a customer's intention to engage in a specific purchasing behavior is determined by their goal to engage in that particular behavioral activity [37]. Numerous empirical studies have demonstrated the value of attitude in understanding behavioral intention, as previously mentioned in psychology, consumer behavior, and marketing. Based on the above, we expect the following:

- ***H7:*** Attitude towards breastfeeding has a positive effect on behavioral intention.

### Actual behavior

Actual behavior has been researched in several marketing fields, including green marketing, luxury brands and products, B2B transactions, and online purchases. According to Ajzen [26], consumer intentions are a sign of how ready consumers are to engage in a particular action, which in this study would be interpreted as actual behavior. Lim et al. [38] remark that behavioral intention and actual behavior must be researched further. Based on the preceding, we hypothesize the following:

- ***H8:*** Behavioral intention towards breastfeeding has a positive effect on actual behavior.

**Fig. 1:**
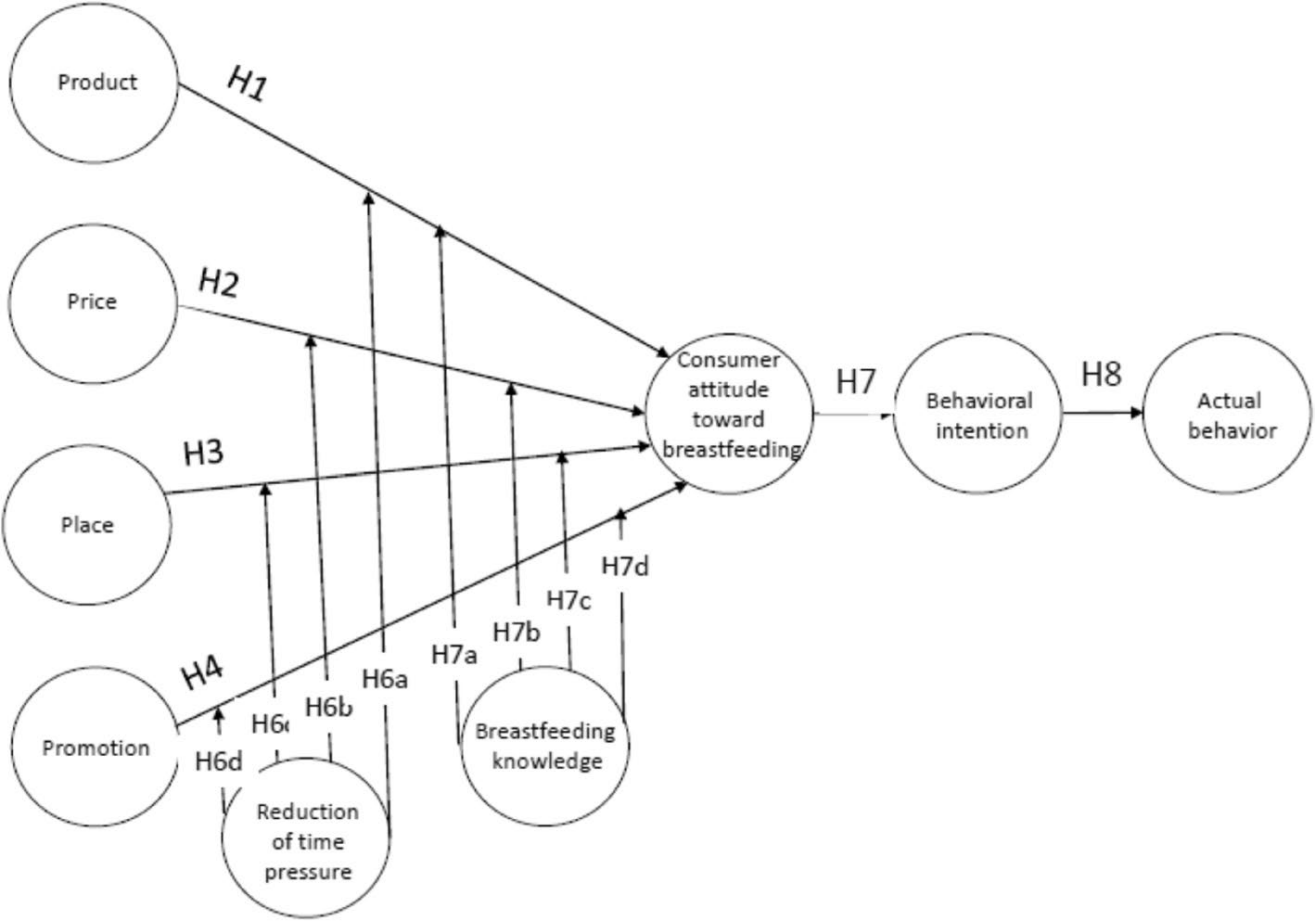
The research model

The study’s conceptual framework is shown in Fig. 1. This model links the demarketing mix of an organization with consumer attitudes, intentions, and behavior toward breastfeeding. It also shows the moderation effect of time pressure reduction and breastfeeding knowledge on each hypothesized path from the demarketing mix variables onto consumer attitudes, intentions, and behavior toward breastfeeding.

## Methods

### Data collection and sample

The paper focuses on implementing demarketing strategies to discourage the consumption of formula milk and promote breastfeeding. The operationalization of demarketing involves identifying the target audience, conducting a situational analysis, developing a demarketing strategy, implementing the planned initiatives, and evaluating their effectiveness. The respondents were breastfeeding mothers in Palestine involved in infant care. They provide insights into their perceptions, knowledge, and attitudes towards breastfeeding and formula milk, helping gauge the impact of the demarketing efforts and refine strategies for promoting breastfeeding. Ultimately, the paper seeks to create a supportive social environment encouraging breastfeeding for improved infant health and well-being.

A descriptive cross-sectional survey research technique was employed in this study. Respondents were encouraged to distribute the questionnaire to breastfeeding mothers in Palestine. The respondents in this paper refer to individuals who participate in the study by providing their responses to the questionnaire. In this case, the respondents are encouraged to distribute the questionnaire to breastfeeding mothers in Palestine. The study employs the snowball sampling technique, where the initial respondents are recruited through breastfeeding support groups, healthcare professionals, and relevant stakeholders who have direct access to breastfeeding mothers. These initial respondents then serve as the starting point for subsequent waves of participants as they share the questionnaire with other breastfeeding mothers within their networks. This approach enables the study to reach a broader and more diverse group of participants, allowing for a comprehensive understanding of breastfeeding promotion and the effectiveness of demarketing strategies in Palestine.

The questionnaire was created specifically for this study using Google Forms to collect primary data and test hypotheses. The first question clarified whether or not the individual is a breastfeeding mother in Palestine. If the participant replied "No," she was told not to access the remainder of the questionnaire. As a result, the study's population includes all breastfeeding women in Palestine. Snowball and convenience sampling were used to choose study participants through personal connections and social media. Every respondent was encouraged to share the survey with their social media contacts. Snowball sampling, also known as chain-referral sampling, is a non-probability sampling approach in which existing subjects are referred to recruit other participants for a research study [39].

The questionnaire was pre-tested by three marketing academics and three marketing practitioners. The researchers then changed the questionnaire based on the feedback they received. The survey was also translated into Arabic (by a professional translator). After two Arabic native speakers confirmed the translation, it was pilot tested for content accuracy. Finally, to alleviate any concerns about participating in the study, the researchers sent them a letter outlining the study goals and inviting them to participate by filling out the questionnaire. The following formula was used to calculate the sample size.

$$N=\frac{NP}{1+\left(NP \times {e}^{2}\right)}$$

With N as the sample size, NP as the population size, and e as the error term (e = 0.05).

According to the calculation, a sample size of 384 was required for a margin error of 5%. No questionnaire was excluded, as the respondent could not submit the questionnaire without answering all the questions. This is because each question is programmed as required to be answered. During the one-month interview period, 369 questionnaires were completed (from September 20, 2021, to October 20, 2021). In the research, it may be necessary to clarify why the sample collection ended before reaching the estimated sample size. The decision to end the sample collection prematurely was influenced by factors such as time constraints, limited resources, logistical challenges, and unforeseen circumstances that made it impractical or unfeasible to continue collecting data until the estimated sample size was reached. We provide transparency and acknowledge any limitations in sample size to ensure the integrity of the research findings and appropriately interpret and generalize the results obtained from the collected data.

The data was analyzed using PLS-SEM modeling with the SmartPLS 3.0 software. A two-step analytic technique was used to analyze the structural equation model. The measuring items were tested for validity and reliability in the first stage. The measurement (outer) model's convergent and discriminant validity were investigated. The square root of the Average Variance Extracted (AVE) was compared to the correlation between latent constructs to test discriminant validity. The structural (inner) model and hypotheses were tested in the second stage. The coefficient of determination was employed to examine the hypotheses' relationships between variables (R2). All hypothesized path coefficients were tested using bootstrapping based on 5,000 resamples.

### Measures

Consumer perceptions of the four demarketing elements were used to determine their effectiveness. First, the product variable was evaluated by a six-item scale adapted from Salem et al. [21]. Second, a five-item scale adapted from Azzam [40] and Salem [1] measured the pricing strategy. Third, the place variable was assessed by a four-item scale adapted from Azzam [40] and Salem [1]. Fourth, the promotion measure was adapted from Salem et al. [41] and Salem [1] using an eight-item scale. The questionnaire's second section includes questions about the moderators. The reduction of time pressure was measured by a four-item scale derived from Van Steenburg & Naderi [42]. Breastfeeding knowledge was assessed by an eleven-item scale derived from Morris et al. [43]. Attitude toward breastfeeding was measured on a five-item scale derived from Yasser Abulreesh et al. [44]. The measure of intention toward breastfeeding was taken from Hsu et al. [45] using a four-item scale. Behavior (actual use) toward breastfeeding was assessed by a three-item scale adapted from Charafeddine et al. [46]. All questions were graded on a seven-point Likert scale ranging from 1 to 5, with 1 indicating “strongly disagree” and 5 meaning “strongly agree.” Various demographic questions were also evaluated, including age, education level, employment status, social class, and monthly income.

## Analysis and calculation

### Descriptive data

The sample demographics are typical of the GS population, as shown in Table 1.

**Table 1:** Profile of consumer respondents (*n* = 369)

Variables	Groupings	No. of respondents	%
Age	25 years or less	80	21.7
	26–35 years	201	54.5
	36 years and above	88	23.8
Education level	High school or lower	65	17.6
	Diploma	70	19.0
	Bachelor	207	56.1
	Master	15	4.1
	Ph.D	12	3.3
Employment status	Full-time employment	74	20.1
	Part-time employment	49	13.3
	Unemployment	238	64.5
	Retirement	8	2.2
Social class	Upper	16	4.3
	Middle	53	14.4
	Lower	265	71.8
	No response	35	9.5
Monthly income	$500 or less	281	76.2
	$501-$1000	61	16.5
	$1001-$1500	8	2.2
	$1501-$2000	3	0.8
	$2001 and above	16	4.3

### Measurement model

Table 2 lists the items on the questionnaire. All construct indicators were combined in the confirmatory factor analysis (CFA) (SmartPLS 3.0) model to assess the measures' psychometric properties. The scale is appropriate for the data, as shown in Table 2. All 55 questions scored higher than 0.70 and were statistically significant at the 0.001 level, suggesting excellent convergent validity. Furthermore, the average variance extracted (AVE) exceeded the minimum criterion of 0.50 in each case. These results support the constructs' convergent validity. The Cronbach's values were all over the 0.70 cut-off level, and the Coefficients of Reliability ranged from 0.702 to 0.962, according to Table 2. These findings indicate that the constructs have high reliability. As indicated in Table 3, discriminant validity was assessed using Fornell and Larcker's [47] criterion, which stipulates that each latent construct's AVE must be larger than the greatest correlation with any other construct. Table 3 reveals that no AVE had a lower correlation with any other component than the maximum correlation, showing discriminant validity.

**Table 2:** Reliability and loading values of the constructs

Construct	Item	Λ	CR	α	AVE
Product	1.1 Decreasing the amount of formula milk packaged in each container	0.722	0.895	0.877	0.586
	1.2 Highlighting the drawbacks of using formula milk as a substitute for breastmilk on the milk container	0.736			
	1.3 Prohibiting the use of infants’ photos as a marketing strategy to promote the sale of formula milk	0.785			
	1.4 Providing statements on the superiority of breastfeeding on formula milk labels based on health professionals' insights	0.771			
	1.5 Explicit warnings on formula milk labels to inform mothers about the risk of contamination of formula milk	0.807			
	1.6 Providing labels illustrating the cost and dangers associated with the unnecessary or improper use of formula milk	0.770			
Price	2.1 Raising the price of formula milk	0.910	0.923	0.895	0.707
	2.2 Raising the tax applied on the (imported) formula milk	0.811			
	2.3 Diminishing cuts on formula milk importing	0.791			
	2.4 Making discounts by the government and other relevant institutions to the women who gave up formula milk	0.787			
	2.5 Increasing the costs associated with importing licenses and/or selling formula milk	0.895			
Place	3.1 Limiting and diminishing the number of places where formula milk is sold	0.821	0.903	0.857	0.700
	3.2 Prohibiting formula milk in private and public health organizations and institutions	0.869			
	3.3 Prohibiting formula milk producers and importers from emphasizing discounts and special displays at the retail level	0.789			
	3.4 Limiting access to formula milk through online and offline channels	0.866			
Promotion	4.1 Prohibiting promotion campaigns for formula milk	0.778	0.921	0.900	0.625
	4.2 Launching campaigns showing the adverse effects of formula milk	0.776			
	4.3 Prohibiting formula milk producers and importers from giving free samples to pregnant women, children, mothers, or health workers	0.793			
	4.4 Prohibiting formula milk producers and importers from exposing pregnant women, children, or mothers to formula milk advertising	0.822			
	4.5 Prohibiting formula milk marketers (company representatives) from making (in)direct contact with or providing advice to pregnant women, children, or mothers	0.828			
	4.6 Prohibiting formula milk producers and importers from launching promotional events supporting formula milk	0.789			
	4.7 Prohibiting health institutions and organizations from promoting and endorsing formula milk	0.746			
	4.8 Prohibiting private and public health organizations from accepting any formula milk samples as advertising material from the producing companies	0.787			
Reduction of time pressure	6.1 Arranging my daily work carefully assists me in breastfeeding my child	0.850	0.873	0.805	0.632
	6.2 I can find time to breastfeed my child if I want	0.783			
	6.3 I can breastfeed my child even if I feel like I am rushing too often	0.814			
	6.4 If I have more free time, I can breastfeed my child	0.728			
Breastfeeding knowledge	7.1 Breastfeeding advantages to the mothers	0.722	0.901	0.874	0.567
	7.2 Breastfeeding advantages to the babies	0.736			
	7.3 Breastfeeding advantages to the family	0.739			
	7.4 Breastfeeding advantages to the environment	0.782			
	7.5 Human milk characteristics	0.713			
	7.6 The risks of the early start of artificial milk	0.779			
	7.7 Anatomy and physiology of breastfeeding	0.782			
	7.8 Breastfeeding technique	0.735			
	7.9 Manual extraction of milk	0.732			
	7.10 Success factors in breastfeeding	0.836			
	7.11 How to cope with breastfeeding hurdles	0.772			
Attitude toward breastfeeding	8.1 Overall, I like the idea of breastfeeding my infant	0.799	0.898	0.858	0.639
	8.2 I do not like to look at different brands of formula milk	0.804			
	8.3 I appreciate the ease of use of breastfeeding	0.790			
	8.4 Breastfeeding is a good thing	0.834			
	8.5 I am positively inclined toward breastfeeding	0.767			
Intentions	9.1 I think I will use breastfeeding in the near future	0.794	0.765	0.749	0.526
	9.2 I have the intention to use breastfeeding	0.828			
	9.3 I would like to use breastfeeding	0.714			
	9.4 I will consider breastfeeding as my first choice	0.747			
Behavior	10.1 I breastfeed my infant many times every day	0.705	0.783	0.762	0.547
	10.2 I depend mainly on breastfeeding to feed my infant	0.777			
	10.3 I breastfeed my infant	0.780

**Table 3:** Discriminant validity of the constructs

	**BE**	**IN**	**P1**	**P2**	**P3**	**P4**	**BK**	**TP**	**ATT**	**BK-ME1**	**BK-ME2**	**BK-ME3**	**BK-ME4**	**RTP-ME1**	**RTP-ME2**	**RTP-ME3**	**RTP-ME4**
BE	0.813																
IN	0.620	0.746															
P1	0.102	0.123	0.765														
P2	0.231	0.215	0.445	0.841													
P3	0.183	0.177	0.568	0.717	0.837												
P4	0.194	0.194	0.523	0.614	0.652	0.783											
BK	0.685	0.614	0.169	0.180	0.161	0.251	0.753										
TP	0.575	0.500	0.170	0.165	0.139	0.272	0.676	0.795									
ATT	0.240	0.253	0.548	0.643	0.700	0.754	0.261	0.274	0.799								
BK-ME1	0.084	0.079	0.040	0.123	0.011	0.042	0.084	0.152	0.043	1.000							
BK-ME2	0.242	0.204	0.098	0.262	0.245	0.212	0.167	0.185	0.264	0.522	1.000						
BK-ME3	0.223	0.171	0.009	0.260	0.210	0.163	0.149	0.177	0.187	0.557	0.805	1.000					
BK-ME4	0.265	0.242	0.035	0.223	0.161	0.195	0.226	0.279	0.168	0.523	0.659	0.752	1.000				
RTP-ME1	0.074	0.091	0.041	0.063	0.045	0.007	0.151	0.152	0.012	0.883	0.411	0.434	0.408	1.000			
RTP-ME2	0.258	0.220	0.054	0.216	0.203	0.174	0.198	0.182	0.231	0.446	0.928	0.760	0.602	0.432	1.000		
RTP-ME3	0.230	0.177	0.040	0.208	0.167	0.127	0.184	0.157	0.144	0.456	0.735	0.923	0.681	0.449	0.786	1.000	
RTP-ME4	0.240	0.213	0.006	0.175	0.125	0.212	0.287	0.317	0.173	0.419	0.572	0.669	0.901	0.419	0.609	0.708	1.000

*P1* Product, *P2* Price, *P3* Place, *P4* Promotion, *RTP* Reduction of Time Pressure, *BK* Breastfeeding Knowledge, *ATT* Attitude toward Breastfeeding, *IN* Intention, *BE* Behavior, *BK-ME1* Breastfeeding Knowledge—Moderating Effect 1, *BK-ME2* Breastfeeding Knowledge—Moderating Effect 2, *BK-ME3* Breastfeeding Knowledge—Moderating Effect 3, *BK-ME4* Breastfeeding Knowledge—Moderating Effect 4, *RTP-ME1* Reduction of Time Pressure—Moderating Effect 1, *RTP-ME2* Reduction of Time Pressure—Moderating Effect 2, *RTP-ME3* Reduction of Time Pressure—Moderating Effect 3, *RTP-ME4* Reduction of Time Pressure—Moderating Effect 4

### Structural model assessment

Table 4 shows the results of the structural model. We employed four alternative models, one without interaction effects and three that examined the interactive impact of each moderating variable individually because SmartPLS can't estimate several moderators simultaneously. The overall fit is suitable across all three models. Furthermore, the composite model's SRMR value for model No. 1 was 0.074, lower than the 0.10 recommended by Bentler [48]. The Stone-Geisser Q2 and the standardized root means residual were utilized to evaluate predictive relevance. Q2 considers the model's and estimated parameters' ability to replicate observed values, with a value larger than 0 showing predictive relevance. The R2 determination coefficient was used to measure the structural model's explanatory power and ability to predict endogenous components. The adjusted R2 value was 0.614, suggesting that the independent factors account for 61.4% of the variation in attitude toward breastfeeding, which is acceptable in social science research. The composite model No. 2 has an SRMR value of 0.079. The adjusted R2 value was 0.652, suggesting that the independent factors explain 65.2% of the variance in attitude toward breastfeeding when the moderating impact of reduced time pressure is considered. The composite model No. 3 has an SRMR value of 0.076. The adjusted R2 value was 0.674, suggesting that the independent factors explain 67.4% of the variation in attitude toward breastfeeding when the moderating impact of breastfeeding knowledge is considered. The magnitude, sign, and significance of path coefficients were all considered. Bootstrapping, which comprised 5,000 resamples drawn with replacement, was used to determine the significance of each estimated path. The findings in Table 4 suggest that all four elements of the demarketing strategy are favorably related to breastfeeding attitudes. In the next section, the results will be examined in further depth.

**Table 4:** Results of structural equation modeling

Dependent variable: Attitude toward Breastfeeding	Model (1)		Model (2)		Model (3)	
**Path model (*n*, model fit indices)**	**Coef**	***t*-value**	**Coef**	***t*-value**	**Coef**	***t*-value**
**(1) Base model (SRMR = 0.097, d_ULS = 3.528, d_G = 1.337. NFI = 0.927)**						
P1	0.170	3.470***	0.164	3.295***	0.163	3.570***
P2	0.233	4.234***	0.230	4.269***	0.229	4.223***
P3	0.159	2.240**	0.149	2.207**	0.137	1.998*
P4	0.369	6.218***	0.341	5.414***	0.357	5.929***
**(2) Interaction model involving reduction of time pressure (SRMR = 0.090, d_ULS = 4.028, d_G = 1.437, NFI = 0.904)**						
RTP			0.147	2.727**		
P1 × RTP			0.138	2.365*		
P2 × RTP			0.142	2.455**		
P3 × RTP			0.126	2.253*		
P4 × RTP			0.016	0.370		
**(3) Interaction model involving breastfeeding knowledge (SRMR = 0.089, d_ULS = 4.738, d_G = 2.121, NFI = 0.915)**						
BK					0.178	2.914***
P1 × BK					0.152	2.537**
P2 × BK					**0.127**	2.301*
P3 × BK					0.134	2.446**
P4 × BK					0.118	2.172*
**Direct effects**	**Coef**			***t*-value**		
Attitude toward breastfeeding—> intention toward breastfeeding	0.631			15.795***		
Intention toward breastfeeding—> behavior toward breastfeeding	0.585			13.415***		

*df* Degrees of freedom, *SRMR* Standardized Root Mean Square Residual, *d_ULS* The squared Euclidean distance, *d_G* the geodesic distance, *NFI* Normed Fit Index, *P1* Product, *P2* Price, *P3* Place, *P4* Promotion, *RTP* Reduction of Time Pressure, *BK* Breastfeeding Knowledge, *ATT* Attitude toward Breastfeeding, *ME1* Moderating Effect 1, *ME2* Moderating Effect 2, *ME3* Moderating Effect 3, *ME4* Moderating Effect 4, *Coef.* Standardized path coefficients

^*^ p < 0.05

^**^ p < 0.01

^***^ *p* < 0.001

## Results and discussion

### Discussion of findings

The four demarketing elements (product, price, place, and promotion) influence consumer attitudes, intentions, and behavior toward breastfeeding. The results prove that the most influential demarketing components on women’s attitudes are, by order of importance, promotion, price, product, and place, supporting H1-4. More specifically, modulating communication efforts, pricing schemes, product reconfigurations, and distribution strategies significantly encourage women to breastfeed infants instead of giving them formula. Furthermore, according to the theory of planned behavior [26], attitudes are also positively linked to intentions, significantly increasing breastfeeding behavior. These results lend support to H7 and H8.

The consistent positive effect of promotion, price, product, and place, respectively, on demarketing under specific consumer states – specifically, reduced time pressure and breastfeeding knowledge – is noteworthy. However, a deeper look into the interactive relationships reveals vital information. More specifically, the results show that time pressure reduction has both a direct and moderating impact on attitudes toward breastfeeding. Although not enhancing the positive impact of the most important demarketing component (i.e., promotion), time pressure reduction strengthens the direct effects of price, product, and place strategies on attitudes, respectively, supporting H5b, H5a, and H5c, but not H5d. In other words, women confronted with a price increase in formula changes in the formula product (e.g., informative labels, warnings, decrease in volume) and lower formula accessibility will be more likely to form a positive attitude toward breastfeeding if they perceive less time pressure. Second, breastfeeding knowledge exerts a similarly direct and interactive effect. However, in contrast to time pressure reduction, breastfeeding knowledge primarily enhances demarketing efforts about product, place, price, and promotion. These results collectively support H6a, H6c, H6b, and H6d, respectively. Consumers with more knowledge about breastfeeding are more likely to develop a positive attitude toward breastfeeding if exposed to demarketing strategies involving product modulations, lower formula accessibility, formula price increase, and communication campaigns prohibiting formula promotion and enhancing breastfeeding. These results support H6a, H6c, H6b, and H6d, respectively.

As confirmed by post hoc LSD tests that followed ANOVA, the effect of the perceived demarketing strategy on reducing breast milk substitutes is significantly different across demographic segments, precisely age [*p* < 0.046], level of education [*p* < 0.008], employment status [*p* < 0.038], social class [*p* < 0.042], and monthly income [*p* < 0.025].

Individuals aged 25 or lower, as well as those aged 26 to 35, are more likely to be influenced by demarketing strategies than those aged 36 and above, according to the LSD test. This might be due to Palestinian society's prevalent customs and traditions, in which the mother-in-law, whose word is generally heard, especially during the early stages of marriage, puts pressure on her daughter-in-law to breastfeed the infant naturally. This may also be because the breastfeeding woman enjoys breastfeeding at a young age, but the novelty effect might attenuate after giving birth to many children. Furthermore, because most of the population is Muslim, the Arab communities (including Palestine) are influenced by Islamic traditions, and several verses in the Holy Quran encourage breastfeeding for the first two years.^1^

The LSD test also revealed that bachelor's and master's degree holders are likelier to be affected by demarketing strategies to encourage breastfeeding than high school or lower degree holders. Furthermore, statistically significant differences existed between diploma holders and master's degree holders, favoring master's degree holders. According to the results, unemployed women are also more likely than full-time employed women to be receptive to demarketing strategies. This is because unemployed women might spend more time at home and face lower time pressure, and the results demonstrated that time pressure reduction significantly enhances demarketing strategies. Furthermore, demarketing strategies do not seem to vary according to social class, but the LSD test revealed that women with lower incomes ($0-$500) are more likely to be affected by demarketing strategies than those with higher monthly incomes ($2,001 and more).

### Theoretical implications

The findings of this study have important implications for advancing the existing theory on breastfeeding promotion, particularly in developing economies. By employing the demarketing framework, the study demonstrates that adjusting product, price, distribution strategies, and communication efforts significantly encourage women to choose breastfeeding over formula feeding.

Furthermore, the study highlights the influence of key moderators, which sets it apart from previous research. Reduced time pressure directly impacts consumer attitudes towards breastfeeding and strengthens the impact of demarketing strategies on product, place, and promotion. Time-constrained individuals may make impulsive purchases and prioritize convenience when shopping. On the other hand, individuals with more free time are less likely to make impulsive purchases. Breastfeeding knowledge follows a similar pattern to reducing time pressure and enhancing the impact of demarketing strategies on product, place, and promotion.

While prior research has focused on price modulations as a critical component of demarketing, this study shows that it is also important to consider promotion, place, price, and product for a better response to encourage breastfeeding.

The study also identifies variations in the effectiveness of demarketing strategies across demographic groups. Younger individuals, particularly those under 36 years old, respond more favorably to demarketing strategies, aligning with previous research highlighting the significance of maternal age in breastfeeding duration. More educated consumers, especially those with university degrees, are also more susceptible to the influence of demarketing strategies, as education positively impacts attitudes towards breastfeeding. Employment status impacts the effectiveness of demarketing strategies, with unemployed women being more influenced than full-time employed women. Workplace commitments and lack of support are significant barriers to breastfeeding for employed mothers. Social class, however, does not appear to significantly impact deconsumption, with women from lower social classes being more receptive to demarketing strategies.

Overall, this study contributes to the literature by shedding light on the effectiveness of demarketing strategies and the moderating role of time pressure, breastfeeding knowledge, demographic factors, and social class. Understanding these implications can inform future research and guide targeted breastfeeding promotion efforts in developing economies.

### Limitations and future research directions

Despite the great care taken in conducting this research, a few limitations are worth mentioning. First, we used non-probabilistic sampling approaches consisting of convenience and snowball sampling. Although we mixed both approaches and conducted face-to-face interviews for better control over the answers and sources of distraction, it might be advisable to perform the analysis using a probabilistic sampling technique to select the respondents. Second, although our study focuses on the demarketing of formula in developing countries, the conclusions drawn for Palestine may not coincide with those about other nations from the Global South. Therefore, additional studies in other developing countries could be advisable. Finally, we only considered two moderators (breastfeeding knowledge and time pressure) for parsimony purposes. Yet, many other moderators could enter into play, such as perceived busyness, the perceived effort of breastfeeding, or perceived wealth, which are commonly explored in socioecological studies (e.g. [49],). All these limitations do not necessarily attenuate the scope of our study, but they surely call for additional replication and comparative studies.

## Data Availability

The datasets used and analyzed during the current study are available from the corresponding author upon reasonable request.

## Footnotes

1 This is noticeable in sura *Luqman,* verse 14: “We have committed man about his parents. His mother carries him in her womb in weakness and frailty, weaning him in two years. So, he should be grateful to Me and to his parents. To Me is the journeying back.”.
